# Prevalence of inappropriate use behaviors of antibiotics and related factors among chinese antibiotic users: an online cross-sectional survey

**DOI:** 10.1186/s12879-022-07671-1

**Published:** 2022-08-13

**Authors:** Xiaoxv Yin, Yanhong Gong, Na Sun, Dandan Li, Jianxiong Wu, Jing Wang, Lei Qiu, Hui Li

**Affiliations:** 1 School of Public Health, Tongji Medical College, Huazhong University of Science and Technology, Wuhan, 430030 People’s Republic of China; 2 International School of Public Health and One Health, Hainan Medical University, Haikou, 570100 People’s Republic of China; 3 School of Health Services Management, Anhui Medical University, Hefei, 230032 People’s Republic of China

**Keywords:** Antibiotic use, Self-medication, Self-storage, Non-adherence, China

## Abstract

**Background:**

Inappropriate use of antibiotics could have a profound negative impact on individual and community. This study aimed to assess the prevalence of inappropriate use behaviors of antibiotics in Chinese antibiotic users and explored their related factors.

**Methods:**

A cross-sectional survey was conducted from July 1, 2018 to September 30, 2018. A structured questionnaire was widely distributed on the online survey platform (Questionnaire Star, https://www.wjx.cn) and was used to collect data from respondents in China on demographic and sociological characteristics, antibiotic use and related knowledge. Main outcomes included self-medication with antibiotics (SMA), self-storage with antibiotics (SSA), and non-adherence to antibiotic treatment (NAAT). Logistic regression was used to identify the related factors of above inappropriate use behaviors of antibiotics.

**Results:**

Of the 15,526 participants, 37.1% reported SMA in the past 6 months, 67.9% reported SSA in the past 6 months, and 48.3%, 15.2%, 25.5% and 78.0% of respondents said that they had missed antibiotics, increased antibiotic dosage, decreased antibiotic dosage, and discontinued antibiotics once symptoms disappear, respectively. Overall, 53.3% reported NAAT during this period. After adjusting for other variables, multivariate logistic regression analyses showed that those aged 30–44 years old, with higher levels of education, poorer status of self-perceived health, or lower levels of antibiotic knowledge were more likely to have these inappropriate use behaviors of SMA, SSA, and NAAT (*P* < 0.05).

**Conclusions:**

The prevalence of SMA, SSA, and NAAT is high in China. Developing a nationwide action plan for the rational use of antibiotics among publics, including national media publicity, online and offline health education, and medication guidance from general practitioners, is urgently needed.

## Key points

1. Self-medication with antibiotics, self-storage with antibiotics, and non-adherence to antibiotic treatment are important performances of inappropriate use behaviors of antibiotics.
2. The results of this study showed that the prevalence of inappropriate use behaviors of antibiotics among Chinese antibiotic users were between 37.1% and 67.9%.
3. A nationwide action plan for rational use of antibiotics is urgently needed in China.

## Background

Under the interaction between supply and demand sides, inappropriate use of antibiotics in humans has attracted widespread attention in the field of global medical and health care [1, 2]. According to the WHO reporting, more than two-thirds of antibiotics were used in community worldwide, about 30.0% of them were used inappropriately [3]. Publics as the main body of antibiotic use, their self-medication with antibiotics (SMA), self-storage with antibiotics (SSA), and non-adherence to antibiotic treatment (NAAT) such as proactively increasing or decreasing drug dosage are important performances of inappropriate use behaviors of antibiotics [4–6]. Such adverse behaviors may lead to negative outcomes including increased risk of antimicrobial resistance (AMR), higher medical costs, failure of treatments, and even death of patients.

The prevalence of inappropriate use of antibiotics reported by different countries varied widely. A sample survey in Europe found that 19.8% of Romanian had SMA, and 68.0% of them had SSA at home [4]. Two surveys in Malaysia and South Korea showed that the prevalence of SMA among residents was between 20.0% and 30.0%, and nearly 55.0% of Malaysian declared they had NAAT, such as discontinuing antibiotics once symptoms disappear [7, 8]. A systematic review from the Gulf Cooperation Council countries showed that the overall prevalence of SMA was about 48.0%, with the highest prevalence in Saudi Arabia (73.0%) [6]. In addition, some demographic and sociological factors including gender, age, education level et al., might be associated with inappropriate use behaviors of antibiotics [8–10]. Huh et al.’s study in South Korea found that residents who were younger or had higher education levels were more likely to have inappropriate use behaviors of antibiotics [8]. However, there were obvious differences in research results in different countries. Therefore, it is necessary to clarify the prevalence of these inappropriate use behaviors and their related factors among antibiotic users in different countries, especially in developing countries.

Inappropriate use of antibiotics is serious in China. Although China has issued a series of policies to promote the appropriate use of antibiotics, most of them focused on limiting antibiotic prescribing in medical institutions and regulating antibiotic for sale in retail pharmacies [11–13]. For example, a 2004 policy stipulated that all retail pharmacies were only permitted to dispense antibiotics with a prescription [13]. In principle, patients cannot buy antibiotics without a doctor’s prescription. However, previous studies showed that about 63%-86% of retail pharmacies in China dispensed antibiotics without a prescription for patients [14, 15]. Non-prescription antibiotic sales at these retail pharmacies increased the risk for self-administering and self-storing antibiotics by publics. For a long time, inappropriate use of antibiotics has been widespread in China. A survey conducted in universities in western China showed that 40.2% of college students had SMA, and more than 56.0% of participants had SSA for future use [16]. A questionnaire survey of 1204 urban residents found that 29.4% of participants had taken SMA, and more than 60.0% of participants had taken SSA [17]. Another survey in rural China found that 62.0% of parents had given SMA for their children without a doctors’ advice [18]. However, previous studies were conducted in individual cities or on specific population, and there was a lack of large national survey of antibiotic use behaviors. Furthermore, the inappropriate use behaviors of antibiotics among publics were diversified, but there was no study on various inappropriate use behaviors and their influencing factors.

Therefore, this study aimed to assess the prevalence of inappropriate use behaviors of SMA, SSA, and NAAT among Chinese antibiotic users, and identified their related factors, in order to provide evidence for the development of targeted intervention strategies.

## Methods

This study was approved by the Ethics Committee of Tongji Medical College, Huazhong University of Science and Technology (Number: 2018IECS175). All participants were informed about the goal of this study and informed consent was obtained from electronic signatures of all participants. Data were collected anonymously. All methods were carried out in accordance with the Declaration of Helsinki.

### Study design and sample size

A cross-sectional survey was designed and conducted among Chinese antibiotic users from July 1, 2018 to September 30, 2018, based on the China’s largest online survey platform (Questionnaire Star, https://www.wjx.cn). Questionnaire Star is a professional service platform for electronic questionnaire design and data collection, which has been widely used by researchers. This study focused on following three aspects of inappropriate use behaviors: SMA, SSA, and NAAT. The NAAT consisted of four types of non-adherence behaviors including missing antibiotics, increasing antibiotic dosage, decreasing antibiotic dosage, and discontinuing antibiotics. To ensure the stability and reliability of the survey results, the sample size should be as large as possible. In our study, the maximum sample size was calculated with the minimum prevalence of these target behaviors. We used the formula:$n={z}^{2}p(1-p)/{d}^{2}$, where *n* is the sample size, *p* is the prevalence of the research target, *z* is the normal deviation (1.96) and *d* is the margin of error (*d* = 0.1*p*). When determining parameter *p*, we took the prevalence of target behaviors among publics in the past 6 months as the criterion according to previous studies. Among them, the prevalence of non-adherence behavior with proactively increasing antibiotic dosage was the lowest with 8.3%–22.0% [17, 19]. Therefore, we took the median value and set the confidence interval (CI) at 95% to calculate the sample size of 2152. Considering the possibility of invalid surveys (about 15%), at least 2532 participants should be surveyed in each region.

According to the China Health Statistical Yearbook (2018) [20], the population who participated in the online questionnaire were divided into three regions: eastern, central and western China. To ensure the representativeness of the survey samples in each region, the administrator would close the network link and end the questionnaire survey when the number of survey samples in each region reached 2532 participants.

### Measurements

To reflect the prevalence of inappropriate use behaviors of antibiotics among Chinese antibiotic users, we selected three main outcomes: the percentage of SMA, the percentage of SSA, and the percentage of NAAT. In this study, SMA was defined as answering “yes” to the question: “During the past 6 months, did you self-administer antibiotics without a doctor’s prescription?” SSA was defined as answering “yes” to the question: “During the past 6 months, did you self-storage antibiotics at home for future use?” NAAT was measured according to four types of non-adherence behaviors that were developed from previous studies [21]: (1) Did you ever miss medication during the course of antibiotic treatment? (2) Did you ever increase the dosage by yourself during the course of antibiotic treatment? (3) Did you ever reduce the dosage by yourself during the course of antibiotic treatment? (4) Once symptoms disappear, do you immediately discontinue the course of antibiotics? Each item measures a type of specific non-adherence behavior, and dichotomous responses (Yes/No) are captured. The item is scored with 0 point if the response is “Yes” and scored with 1 point if the answer is “No”. The total scores range from 0 to 4, with the higher the score, the higher the antibiotic user’s adherence. The total scores ≤ 2 are considered as “non-adherence”, and the total scores > 2 are considered as “adherence.”

To identify the relevant factors of inappropriate use behaviors of SMA, SSA, and NAAT, we also collected the demographic and sociological characteristics, including gender, age, place of residence (urban/rural), educational level (junior high school or below/ high school/junior college or above), self-perceived economic status (good/ average/poor), self-perceived health status (good/ average/poor), and antibiotic knowledge. Antibiotic knowledge is assessed by asking 10 questions in three sections: the knowledge regarding antibiotic role, antibiotic use and antibiotic resistance. These 10 questions are estimated using a scoring scheme, with score of 1 for a correct response and 0 for an incorrect response. The total scores of knowledge range between 0 and 10. We divided the total scores of antibiotic knowledge into three levels as following: (1) high level: 8–10 scores; (2) medium level: 3–7 scores; (3) low level: 0–2 scores. In the questionnaire, Cronbach’s α coefficients of non-adherence behavior scale and antibiotic knowledge scale were 0.71 and 0.84 (> 0.70), respectively. Confirmatory factor analyses showed that the standardized factor loading of each item of non-adherence behavior scale ranged from 0.52 to 0.57 (> 0.40), and these values of the antibiotic knowledge scale were all above 0.70. The results of these tests indicated that the questionnaire had good reliability and validity.

### Data collection

We designed a questionnaire and generated a valid QR code of this questionnaire on the online survey platform. Then, we recruited 50 graduate and undergraduate students from the School of Public Health at Huazhong University of Science and Technology as investigators in July 2018. Four of them refused the request to distribute questionnaires, with a participation rate of 92%. After the unified training, the investigators would get the QR code of questionnaire. During the summer vacation, all sent out electronic questionnaires through their personal social software such as “WeChat”. The respondents scanned the QR code on their mobile phones to fill in the questionnaire. The data administrator checked the questionnaire and sorted out the data through the background every week. When each region (eastern, central and western China) reached the minimum required survey sample of 2532, the administrator closed the network link and finished the survey.

Before the survey, we conducted a questionnaire pilot test to ensure the smooth implementation of all steps including the respondents’ login on the platform, questionnaire filling and submission. we set the following inclusion criteria that the respondents filling in the questionnaire must meet: (1) adults aged 18 years or above; (2) living in the area for more than 6 months; (3) normal cognitive and understanding abilities; (4) aware of the term “antibiotic” and having used antibiotics. To ensure that only one questionnaire per respondent can be filled out, we limited one IP address per submission on the online survey platform and required to log in with a personal mobile phone number (in China, the mobile phone number is real-name system) before filling in the questionnaire. During the survey, to improve the enthusiasm of respondents, we paid 3 Yuan in cash to each person who completed the questionnaire as an economic incentive. To ensure that respondents must read and answer the questionnaire carefully, we set three quality inspection questions at different locations, namely: “Where is the capital of China?”, “What is 7 minus 2?”, and “What is 1 plus 3?” If the answer to any of these three questions was incorrect, the questionnaire would be marked as invalid. After the respondents completed the questionnaires, all the questionnaires were automatically input into a data file, and two researchers independently collated and verified the data to ensure the reliability of the survey data.

During the process of the survey, 21,874 respondents visited the questionnaire link, and 17,062 respondents actually completed the questionnaire. Since this was an online survey, the true response rate could not be determined here. Among all the questionnaires, 1280 questionnaires were excluded because of unqualified quality inspection, and 256 questionnaires were also excluded because of respondents under the age of 18. Finally, 15,526 valid questionnaires were obtained.

### Statistical analysis

Descriptive analysis of the demographic and sociological characteristics, antibiotic knowledge, and various inappropriate use behaviors of the participants was conducted. The classified variables were expressed as frequency and percentage, and the continuous variables were expressed as mean and standard deviation (SD). To examine the possible impact of the controversial Q5 and Q9 from the antibiotic knowledge scale (Table 2) on the study results [22, 23], a sensitivity analysis was performed by deleting these two items. When these two items were removed, the total scores for antibiotic knowledge became between 0 and 8. The total scores of antibiotic knowledge were re-divided into three levels as following: (1) high level: 7–8 scores; (2) medium level: 3–6 scores; (3) low level: 0–2 scores. Furthermore, binary logistic regression model was used to explore the factors associated with the inappropriate use behaviors of SMA, SSA, and NAAT among antibiotic users. Adjusted odds ratio (aOR) and 95% CI for each variable were given. All analyses were performed using SAS 9.4 (SAS Inc., Cary, NC). Statistical tests were double-tailed; a *P* value < 0.05 was considered to be significant.

## Results

### Characteristics of participants

Of the 15,526 valid participants, 6222 (40.1%) were male. Their mean age was 34.6 (SD = 11.7) years old. Among all the participants, 70.8% were from urban places of residence, 64.3% had a junior college education or higher, more than 75.0% of participants self-perceived their economic status as average, and nearly half (49.1%) of participants self-perceived their health status as good (Table 1).

**Table 1:** Demographic and sociological characteristics of participants

Variables	Items	Number	Percentage (%)
Gender	Male	6222	40.1
	Female	9304	59.9
Age (years old)	18–29	6347	40.9
	30–44	5262	33.9
	≥ 45	3917	25.2
Place of residence	Urban	10,991	70.8
	Rural	4535	29.2
Region in China	Eastern	2293	14.8
	Central	6489	41.8
	Western	6744	43.4
Education level	Junior high school or below	2342	15.1
	High school	3207	20.7
	Junior college or above	9977	64.3
Self-perceived economic status	Good	1808	11.6
	Average	11,672	75.2
	Poor	2046	13.2
Self-perceived health status	Good	7620	49.1
	Average	6845	44.1
	Poor	1061	6.8

### Antibiotic knowledge of participants

Of all the participants, more than 55.0% could not correctly identify whether antibiotics were used to treat bacterial or viral infections, and 57.6% could not distinguish the concept of antibiotics and anti-inflammatory agents. Regarding the knowledge of antibiotic use, 50.8% believed they could change the antibiotic dosage by themselves and nearly 60.0% believed that they could immediately discontinue the course of antibiotics once the symptoms disappeared. Regarding the knowledge of antibiotic resistance, 53.5% were not aware of the increased risk of resistance if not following a course of antibiotics. Overall, 26.0%, 46.6% and 27.4% of the participants had high, medium, and low levels of antibiotic knowledge, respectively (Table 2). By deleting Q5 and Q9, the sensitivity analysis showed that the proportion of participants with high, medium and low levels of antibiotic knowledge was 21.3%, 45.1% and 33.6%, respectively.

**Table 2:** Antibiotic knowledge of participants

Items	Incorrect response	
	Number^a^	Percentage (%)
Knowledge regarding antibiotic role		
Q1 Antibiotics can treat bacterial infections	8808	56.7
Q2 Antibiotics can treat viral infections	9594	61.8
Q3 Antibiotics are the same as anti-inflammatory agents	8941	57.6
Knowledge regarding antibiotic use		
Q4 Antibiotic must be purchased with a doctor’s prescription	6577	42.4
Q5 You can take many kinds of antibiotics at the same time during the course of a single illness	7410	47.7
Q6 You can change the antibiotic dosage by yourself during the course of antibiotic treatment	7883	50.8
Q7 You can immediately discontinue the course of antibiotics once symptoms disappear?	9270	59.7
Knowledge regarding antibiotic resistance		
Q8 Antibiotic overuse can result in antibiotic resistance	5802	37.4
Q9 Non-adherence to the treatment course of antibiotics would increase the risk of antibiotic resistance	8311	53.5
Q10 Antibiotic resistance is a serious hazard to people	6266	40.4
Knowledge level^b^		
High	4037	26.0
Medium	7240	46.6
Low	4249	27.4

^a^They are true or false questions, with Q2, Q3 and Q5-Q7 correctly respond as “false”; Q1, Q4 and Q8-Q10 correctly respond as “true”

^b^The total scores of knowledge range between 0 and 10. The total scores of antibiotic knowledge are divided into three levels as following: (1) high level: 8–10 scores; (2) medium level: 3–7 scores; (3) low level: 0–2 scores

### Prevalence of inappropriate use behaviors of antibiotics

Of the 15,526 respondents, 37.1% reported SMA in the past 6 months, and 67.9% reported SSA in the past 6 months. Regarding the four types of non-adherence behaviors, 48.3%, 15.2%, 25.5% and 78.0% of respondents said that they had missed antibiotics, increased antibiotic dosage, decreased antibiotic dosage, and discontinued antibiotics once symptoms disappear, respectively. Overall, 53.3% had NAAT during this period (Table 3).

**Table 3:** The prevalence of self-medication with antibiotics, self-storage with antibiotics, and non-adherence to antibiotic treatment

Variables	Number	Percentage (%)
Self-medication with antibiotics (SMA)	5766	37.1
Self-storage with antibiotics (SSA)	10,539	67.9
Types of non-adherence behaviors^a^		
(1) Missing antibiotics	7495	48.3
(2) Increasing antibiotic dosage	2361	15.2
(3) Decreasing antibiotic dosage	3960	25.5
(4) Discontinuing antibiotics early	12,104	78.0
Non-adherence to antibiotic treatment (NAAT)^b^	8271	53.3

^a^Each item measures a type of specific non-adherence behavior and the number of “Yes” answer for each item is identified

^b^The total scores range from 0 to 4, with the total scores ≤ 2 are considered as “non-adherence”, and the total scores > 2 are considered as “adherence.”

### Factors associated with inappropriate use behaviors of antibiotics

The results of multivariate logistic regression analyses showed that respondents aged 30–44 years old (aOR = 1.36, 95% CI 1.26–1.47), ≥ 45 years old (aOR = 1.25, 95% CI 1.15–1.37), with junior college or above level of education (aOR = 1.16, 95% CI 1.05–1.29), with average (aOR = 1.41, 95% CI 1.32–1.52) or poor (aOR = 1.57, 95% CI 1.38–1.80) status of self-perceived health, with medium (aOR = 1.08, 95% CI 1.02–1.15) or low (aOR = 1.40, 95% CI 1.28–1.54) levels of antibiotic knowledge had a higher risk of SMA. Those who were female (aOR = 1.21, 95% CI 1.13–1.30), aged 30–44 years old (aOR = 1.41, 95% CI 1.29–1.53), with high school (aOR = 1.26, 95% CI 1.12–1.41) or junior college or above (aOR = 1.54, 95% CI 1.39–1.71) levels of education, with average (aOR = 1.28, 95% CI 1.19–1.38) or poor (aOR = 1.36, 95% CI 1.18–1.58) status of self-perceived health, with medium (aOR = 1.10, 95% CI 1.01–1.20) or low (aOR = 1.16, 95%CI 1.06–1.28) levels of antibiotic knowledge were more likely to have SSA. Respondents aged 30–44 years old (aOR = 1.43, 95% CI 1.32–1.55), ≥ 45 years old (aOR = 1.60, 95% CI 1.47–1.75), with high school (aOR = 1.16, 95% CI 1.03–1.30) or junior college or above (aOR = 1.30, 95% CI 1.18–1.45) levels of education, with average (aOR = 1.21, 95% CI 1.13–1.30) or poor (aOR = 1.20, 95% CI 1.05–1.38) status of self-perceived health, with medium (aOR = 1.57, 95% CI 1.45–1.70) or low (aOR = 2.06, 95%CI 1.88–2.26) levels of antibiotic knowledge were more likely to have NAAT (Table 4).

**Table 4:** Multivariate logistic regression analysis of self-medication with antibiotics, self-storage with antibiotics, and non-adherence to antibiotic treatment ^b^

Variables	Self-medication	Self-storage	Non-adherence
	aOR^a^ (95% CI)	aOR^a^ (95% CI)	aOR^a^ (95% CI)
Gender (Ref = Male)			
Female	1.00 (0.94–1.07)	1.21 (1.13–1.30)***	1.07 (1.00–1.14)
Age (Ref = 18–29)			
30–44	1.36 (1.26–1.47)***	1.41 (1.29–1.53)***	1.43 (1.32–1.55)***
≥ 45	1.25 (1.15–1.37)***	1.08 (0.98–1.18)	1.60 (1.47–1.75)***
Place of residence (Ref = Urban)			
Rural	1.00 (0.93–1.08)	0.86 (0.79–0.93)***	1.02 (0.94–1.10)
Region in China (Ref = Eastern)			
Central	0.92 (0.84–1.02)	0.97 (0.87–1.07)	0.95 (0.86–1.05)
Western	1.04 (0.94–1.14)	1.08 (0.97–1.19)	0.97 (0.88–1.07)
Education level (Ref = Junior high school or below)			
High school	1.04 (0.93–1.17)	1.26 (1.12–1.41)***	1.16 (1.03–1.30)*
Junior college or above	1.16 (1.05–1.29)**	1.54 (1.39–1.71)***	1.30 (1.18–1.45)***
Self-perceived economic status (Ref = Good)			
Average	0.79 (0.71–0.87)***	0.90 (0.80–1.01)	0.95 (0.86–1.06)
Poor	0.66 (0.57–0.76)***	0.69 (0.59–0.80)***	0.94 (0.82–1.08)
Self-perceived health status (Ref = Good)			
Average	1.41 (1.32–1.52)***	1.28 (1.19–1.38)***	1.21 (1.13–1.30)***
Poor	1.57 (1.38–1.80)***	1.36 (1.18–1.58)***	1.20 (1.05–1.38)**
Knowledge level (Ref = High)			
Medium	1.08 (1.02–1.15)*	1.10 (1.01–1.20)*	1.57 (1.45–1.70)***
Low	1.40 (1.28–1.54)***	1.16 (1.06–1.28)**	2.06 (1.88–2.26)***

^a^When one of the variables is analyzed, the other variables are adjusted as covariates and aOR is the adjusted odds ratio

^b^The P values of the likelihood ratio test of the logistic regression models of self-medication with antibiotics, self-storage with antibiotics, and non-adherence to antibiotic treatment are all less than 0.05, so it could be considered that three fitted models are statistically significant

*P < 0.05, **P < 0.01, ***P < *0.001*

## Discussion

This study investigated the inappropriate use behaviors of antibiotics and explored their related factors among 15,526 Chinese antibiotic users, and the results showed that the prevalence of SMA, SSA, and NAAT was common in China, and these inappropriate antibiotic behaviors had some similar related factors.

Our study found that the prevalence of SMA was 37.1% in the past 6 months. Globally, this rate was close to or higher than that in some low- and middle-income countries, such as Kenya (45.1%) [24] and Serbia (27.2%) [25], and well above most high-income countries, such as the UK (5.0%) [26]. SSA is another important performance of inappropriate use behaviors of antibiotics. This study showed that the prevalence of SSA was 67.9% in the past 6 months. It was significantly higher than that in low- and middle-income countries, such as Serbia at 49.1% [25] and Jordan at 18.4% [27]. Furthermore, the prevalence of NAAT was 53.3% in our study. Due to inconsistent methods of measuring non-adherence behaviors, the overall prevalence of NAAT was difficult to compare with previous studies. In spite of this, 78.0% of participants immediately discontinued antibiotics once the symptoms disappeared, which was also higher than that in Saudi Arabia (71.1%) [28]. Compared with clinicians’ antibiotic prescribing behavior, patients’ self-storage with antibiotics at home, self-medication with antibiotics when ill, and increasing or reducing the dosage by themselves during the course of antibiotic treatment et al., were more likely to lead to the increased risk of AMR [2, 29]. If the patients’ disease would be handled properly, it could further lead to hospitalization, secondary infection, and even death [30]. Therefore, the above results indicated the urgent need for a national action plan and effective public health strategies to address the widespread inappropriate use behaviors of antibiotics in China.

This study comprehensively explored the related factors of inappropriate use behaviors of SMA, SSA, and NAAT. The results showed that age, education level, self-perceived health status and antibiotic knowledge level were associated with all three inappropriate use behaviors. The population aged 30–44 years old had the highest risk of SMA and SSA. This may be due to the fact that most aged 30–44 years old are in the ascendance of their careers and the time cost of seeking medical services in hospitals is too high, which may lead them to choose SMA and SSA [31]. It was worth noting that the increase of participants’ education level did not have a positive effect on appropriate use of antibiotics. Conversely, participants’ with higher levels of education were more likely to have these inappropriate use behaviors. This was consistent with studies in Greece and Italy [32, 33], in which the researchers explained that residents with higher levels of education were more likely to believe they can appropriately use antibiotics, so they overestimated their understanding of antibiotic use. Participants with poorer status of self-perceived health were more likely to have these inappropriate use behaviors. It was similar to findings from countries such as Brazil, Italy and New Zealand [34–36]. Indeed, the population with poorer status of self-perceived health take medication frequently even if unnecessary for their condition, and long-term medication habits make them tend to rely on medication, so as to increase the risk of inappropriate use of antibiotics [34].

In this study, the overall level of antibiotic knowledge in Chinese antibiotic users was not high, and the lower the level of antibiotic knowledge, the higher the risk of these inappropriate use behaviors. Many health behavior change theories, such as Health Belief model and Knowledge-Attitude-Practice model, agree that knowledge is the prerequisite and important basis for behavior change and also provides necessary information for individuals to make health decisions [37, 38]. Therefore, carrying out health education is necessary. Based on scientific knowledge transformation theories, researchers transfer the related antibiotic knowledge to the target population through appropriate channels, so as to change inappropriate use behaviors of antibiotics among publics.

This study was the first to conduct a survey of inappropriate use behaviors of antibiotics in a large sample of antibiotic users in central, eastern and western China. As this survey was conducted online, 74.8% of participants aged 18–44 years old and 64.3% of participants had junior college or above. Combined with the above association between age and education level and inappropriate use behaviors of antibiotics, the lack of elderly and non-educated population might lead to a higher prevalence in this study. On the whole, our population was young and highly educated, but their level of antibiotic knowledge was still low. Therefore, online health education on the rational use of antibiotics should be a priority strategy for young and middle-aged population. In addition, by removing Q5 and Q9, the sensitivity analysis showed that although the proportion of participants with different levels of antibiotic knowledge had changed, the overall level of antibiotic knowledge in Chinese antibiotic users was still not high, and there was still a significant negative association between antibiotic knowledge level and these inappropriate use behaviors. This proved the stability of research results to some extent. At present, AMR has become a global public health problem. WHO is urging all countries to participate in the *Global Action Plan on Antimicrobial Resistance* to promote the rational use of antibiotics and reduce the AMR [39]. Our study timely focuses on the inappropriate use behaviors of antibiotics in China, a populous country, which is of great significance for the control of global AMR.

However, our study had several limitations. Firstly, as with all self-reported questionnaire survey, the results were inevitably subject to recall bias. Secondly, this study adopted the form of online questionnaire to carry out the survey. As young people were more skilled in using mobile phones and social software, the population in this study was young, highly educated and has good self-reported health, which might lead to a certain degree of response bias, namely a higher prevalence. Thirdly, this study was a cross-sectional survey. Therefore, it was difficult to infer causal conclusions, and further prospective studies should be conducted to test the conclusions of this study.

## Conclusions

Under the characteristics of young and highly educated population, the overall level of antibiotic knowledge in Chinese antibiotic users is still low, and the prevalence of SMA, SSA, and NAAT was at a high level. Those aged 30–44 years old, with higher levels of education, poorer status of self-perceived health, or lower levels of antibiotic knowledge were more likely to have all three inappropriate use behaviors of antibiotics. In the future, it is very necessary for the Chinese government to formulate a nationwide action plan for the rational use of antibiotics, especially for young and middle-aged population, including national media publicity, online and offline health education, and medication guidance from general practitioners, so as to promote the rational use of antibiotics in the daily life of the publics.

## Data Availability

The full datasets are available through the corresponding author upon reasonable request.

## Abbreviations

SMA: Self-medication with antibiotics
SSA: Self-storage with antibiotics
NAAT: Non-adherence to antibiotic treatment
WHO: World health organization
AMR: Antimicrobial resistance
CI: Confidence interval
aOR: Adjusted odds ratio
SD: Standard deviation
